# Slit2/Robo1 Mitigates DSS-induced Ulcerative Colitis by Activating Autophagy in Intestinal Stem Cell

**DOI:** 10.7150/ijbs.42331

**Published:** 2020-04-06

**Authors:** Jingzhou Xie, Li Li, Shuhua Deng, Jiayuan Chen, Quliang Gu, Huanhou Su, Lijing Wen, Sheng Wang, Caixia Lin, Cuiling Qi, Qianqian Zhang, Jiangchao Li, Xiaodong He, Weidong Li, Lijing Wang, Lingyun Zheng

**Affiliations:** 1 School of Life Sciences and Biopharmaceutics, Guangdong Pharmaceutical University, Guangzhou 510006, Guangdong, PR China.; 2 Institute of Health, Guangdong Pharmaceutical University, Guangzhou 510006, Guangdong, P. R. China.; 3 Guangdong Engineering Research Center for Light and Health, Guangdong Pharmaceutical University, Guangzhou 510006, Guangdong, P. R. China.

**Keywords:** Slit2/Robo1 signal, UC, autophagy, Lgr5 stem cells

## Abstract

Ulcerative colitis (UC) is a recurrent intestinal inflammatory disease. Slit2, a secreted protein, interacts with its receptor Robo1 to regulate the differentiation of intestinal stem cells and participate in inflammation and tumor development. However, whether Slit2/Robo1involved in the pathogenesis of UC is not known. We investigated Slit2/Robo1-mediated UC using a dextran sodium sulfate (DSS)-induced model. Eight-week-old male Slit2-Tg (Slit2 transgene) mice, Robo1/2^+/-^ (Robo1^+/-^ Robo2^+/-^) mice, and their WT littermates were allocated into two groups: (I) control group (n=10), of mice fed a normal diet and tap water and (II) DSS group (n=10), of mice fed a normal diet and drinking water with 2% DSS for 7 days. Colon tissues were collected and analyzed by qPCR, immunohistochemistry, western blot, and immunofluorescence. Slit2-Tg DSS mice showed less body weight loss, less blood in the stool, and less viscous stool compared to those of WT_Slit_ DSS mice. Robo1/2^+/-^ DSS mice displayed a heavier degree of blood in the stool and a more apparent viscosity of the stool compared to those of WT_Robo1/2_ DSS mice. Slit2 overexpression maintained Lgr5^+^ stem cell proliferation in the crypt after DSS treatment, significantly increased the LC3II/I ratio, and slightly stimulated p62 expression in the crypt compared to those of DSS-induced WT_Slit_ mice. Robo1/2 partial knockout reduced the number of Lgr5^+^ stem cells, decreased the LC3II/I ratio, and markedly increased p62 expression in the crypt compare to those of DSS-treated WT_Robo1/2_ mice. Our findings suggest that Slit2/Robo1 mediates DSS-induced UC probably by activating the autophagy of Lgr5^+^ stem cells.

## Introduction

Ulcerative colitis (UC) is a nonspecific intestinal inflammatory disease with unknown etiology that is mainly characterized by diarrhea, abdominal pain, mucus pus, and bloody stool 1. Accumulated evidence shows cytokine-producing lymphocytes, plasma cells, and macrophages and neutrophils infiltrated in the colonic mucosa of UC patients 2. Moreover, proinflammatory cytokines such as IL-1, IL-6, IL-8, and tumor necrosis factor (TNF) are involved in the pathogenesis of UC 3.

Initiation of the inflammatory reaction mainly occurs through the innate immune system using the products encoded by germline gene- pattern recognition receptors (PRRs) to recognize pathogen-associated molecular patterns (PAMPs) or injury-related molecular patterns 4, 5. Cells recognize exogenous or endogenous ligands through toll-like receptors (TLRs) and nucleotide oligomerization domain (NOD)-like receptors (NLRs), leading to receptor activation, which in turn leads to a signal transduction cascade of multiple proteins, secretion of proinflammatory factors and activation of the adaptive immune response. Recent studies have shown that a variety of TLR ligand PAMPs can induce autophagy, which has an obvious negative regulatory effect on TLR signaling and the inflammatory response 6, 7. Notably, dysfunctional autophagy in the intestine results in inflammation and contributes to an increased susceptibility to Crohn's Disease 8. Some evidence suggests that restoring impaired autophagy could alleviate inflammation in the intestinal diseases 9.

Autophagy is a self-protective mechanism for maintaining homeostasis and is an evolutionarily conserved process that begins by the formation of a double-membrane autophagosome engulfed cytoplasmic contents. The final step of the autophagy process is the degradation and the recycling of the enwrapped materials by lysosomal enzymes following the fusion of autophagosomes with lysosome 10-12.

The critical feature in the process is the formation of autophagosomes, which involve a set of autophagy-related (ATG) proteins in a hierarchical manner. Firstly, recruitment of ATG9 and mATG8s including LC3 and GABARAP subfamilies promotes the expansion of the phagophore membrane 13; secondly, conjugation of LC3s to the membrane-resident lipid phosphatidylethanolamine (PE) is modified by the protease ATG4, the E1 enzyme ATG7, the E2 enzyme ATG3, and the E3 ligase complex including ATG5/ATG12/ATG16L1 14; thirdly, insertion of modified LC3s in the phagophore complete the elongation and maturation of autophagosome. Furthermore, LC3 interacts with cargo receptors, such as the sequestosome1/p62-like family proteins, which confer selectivity to autophagic degradation after autophagosome fuse with the lysosome 15.

The intestinal mucosa is one of the most active places in the mammalian body 16. Intestinal epithelial cells are continuously self-renewing because the stem cells located in the intestinal crypt possess a strong ability to proliferate and differentiate into absorption cells, goblet cells, endocrine cells and Paneth cells, all of which not only maintain normal physiological functions but also repair damaged intestinal epithelium 16-19. Although several reviews have shown that stem cell self-renewal and differentiation depend on the activation of autophagy 20, 21, whether the autophagy of intestinal stem cell involves in the intestinal barrier and mucosal homeostasis is unknown.

Slit1-3 are glycoproteins that are expressed in the nervous system and immune system 22. Slit can bind to the Roundabout receptor (Robo1, 2, 3, and 4) to activate Slit/Robo signaling 23, 24. Slit2 protein can significantly slow the recruitment of leukocytes from rat lymph nodes, to the inflammatory site by inhibiting the chemotaxis of these cells to chemokines such as SDF-1 25. Notably, in the small intestine, Slit2 and Robo1 are specifically expressed in Lgr5^+^ stem cells in the intestinal recess and promote the differentiation of Lgr5^+^ stem cells into epithelial cells to maintain the integrity of the intestinal tract 26, indicating that Slit2-Robo1might regulate autophagy in the Lgr5^+^ stem cells.

Therefore, in this study, we explored the role of Slit2/Robo1 in the autophagy of intestinal Lgr5^+^ stem cells during the development of UC.

## Materials and Methods

### Animals and Treatment

Slit2-Tg mice on a C57BL/6J background obtained from the lab of JianGuo Geng. Their age-matched wild-type littermates WT_Slit_ mice were purchased from the Guangdong Medical Laboratory Animal Center. Robo1/2^+/-^ mice and their WT littermates (Robo1^+/+^ Robo2^+/+^mice, WT_Robo1/2_ mice) were obtained from the lab of JianGuo Geng. All mice were maintained on a 12 h light/dark cycle with controlled constant temperature (24 ± 2°C) and humidity (60 ± 5%). Use and experiments on the animals in this study were in accordance with the ethics approval of Guangdong Pharmaceutical University. Eight-week-old male mice of each genotype were randomly assigned to two groups (n=10): (1) control group, mice were fed normal diet and distilled water; (2) DSS group, mice were given normal diet and 2% DSS in distilled water for 7 consecutive days. Clinical parameters, such as weight loss, diarrhea and rectal bleeding, were monitored daily.

### Histology

Intestinal tissues fixed in 4% buffered paraformaldehyde overnight were embedded in paraffin. Sections with a thickness of 3 μm were stained with H&E for routine histology examination. Photomicrographs were obtained using a microscope (OLYMPUS, Japan).

### Quantification methods of histological score, stool consistency index and fecal blood index

At necropsy, the colons of the mice were dissected and lengths were measured by plate gage. Blinded histological scores were assigned using validated scales. The colon scores are as follows: 0, no inflammation; 1, low inflammation with scattered infiltrating cells (1-2foci); 2, moderate inflammation with multiple foci (with epithelial hyperplasia and mild loss of goblet cells); 3, high inflammation with increased vascular density and marked wall thickening (with obvious epithelial hyperplasia and goblet cell depletion); and 4, maximal inflammation (with transmural leukocyte infiltration and loss of goblet cells). Fecal blood was assayed with a Hemoccult test (Baso, Zhuhai, China) according to the following scale: 0, no color; 1, purple-red changes gradually (within 1-2min); 2, Fuchsia (within 1min); 2.5, purple-blue (within 10s); 3, purple-blue (1s); and 4, gross bleeding. Stool consistency was quantified as follows: 0, normal; 1, pasty; 2, soft, but formed; 3, soft, no form; and 4, diarrhea.

### Immunohistochemistry (IHC)

In brief, paraffin sections were deparaffinized, rehydrated, immersed in 3% hydrogen peroxide for 30 mins to inactivate any endogenous peroxidase and then incubated for 1 h in blocking buffer containing 10% bovine serum albumin (BSA). After blocking, the sections were incubated overnight at 4°C with the following primary antibodies: anti-ki67(1:100, Abcam) and anti-Lgr5(1:100, Origen). After washing with PBS three times, the sections were incubated at 37°C with horseradish peroxidase (HRP)-conjugated anti-rabbit secondary antibody. Images of nine randomly selected fields were acquired under a microscope (OLYMPUS, Japan). The protein expression levels in the slices were quantified using Image-Pro Plus 4.5 software (Media Cybernetics, MD, USA).

### Immunofluorescence

After dehydration, the tissue was embedded and cooled at 4°C. After repair, the tissue was sliced at a thickness of 3 μm and heated at 65°C for 30 mins. After dewaxing, the section was washed with PBS three times and repaired in sodium citrate (pH = 6.0) for 10 mins. Then, the section was blocked with 10% BSA for 1 h and incubated with the primary antibody overnight. The following antibodies were used for primary antibody incubation: anti-BrdU (1:100, Zsbio), anti-Lgr5 (1:100, Origen), and anti-LC3 (1:100, CST). The section was further incubated in a secondary antibody at 37°C for 1 h. After washing with PBS three times, the section was stained with hematoxylin for 3 min and sealed for photographing.

### Real-time PCR

Total RNA was isolated from colorectum using Trizol reagent (Invitrogen, USA) following the manufacturer's instructions. The RNA concentration in each sample was measured using a NanoDrop ND-1000 (Thermo Fisher Scientific, MA, USA), and complementary DNA was synthesized by PrimeScript RT Master Mix Perfect Real-Time Kit (TaKaRa, Naha, Japan). The specific primers were designed and synthesized by Sangon Biotech, and the quantitative values of the genes in the samples were normalized using GAPDH as the internal control. Primer sequences were: IL-6 (forward: 5'AGGATACCACTCCCAACAGA3' and reverse: 5'ACTCCAGGTAGCTATGGTACTC3'), TNF-α (forward: 5'CTGAACTTCGGGGTGATCGG3' and reverse: 5'GGCTTGTCACTCGAATTTTGAGA3'), Lgr5 (forward: 5'CTTCACTCGGTGCAGTGCT3' and 5'GTACTGCCGTGGTCCACAC 3'), GAPDH (forward: 5'AGGTCGGTGTGAACGGATTTG3' and reverse: 5'TGTAGACCATGTAGTTGAGGTCA3'). The fold-increase over the internal control values was determined using the relative quantification method of 2^-△Ct^.

### Crypt isolation

Briefly, the colons from 8-week-old mice of each group were opened longitudinally, washed 3× with cold PBS and cut into 1-2 mm pieces. The tissues were incubated with 2 mM EDTA in cold PBS for 30 min at 4 °C on a rotating wheel. After centrifugation at 400 × g for 2min at 4 °C, the supernatant containing villi was removed and incubated in cold PBS/EDTA (2 mM) for 30 min at 4 °C on a rotating wheel. This procedure was repeated three times. Crypts were then detached from the basal membrane via moderate shaking to preserve the intact crypt structures. The crypts enriched in the supernatant were passed through a 70-μm cell strainer (BD Biosciences, Heidelberg, Germany), centrifuged at 4 °C and 250 × *g* for 3 min and resuspended in PBS. The purity of crypt preparations was check by microscopy.

### Western Blotting

The total cell proteins of the colon crypts were extracted using RIPA buffer and separated using 15% and 10% (w/v) Sodium dodecylsulphate-polyacrylamide gel electrophoresis (SDS-PAGE) before being transferred onto a polyvinylidene fluoride (PVDF) membrane (Millipore, MA, USA). The membranes were blocked using 5% (w/v) non-fat powder milk for 1 h at room temperature. Rabbit anti-mouse LC3 polyclonal antibody (1:500, ABclonal), rabbit anti-mouse p62 polyclonal antibody (1:500, Affinity Biosciences), and mouse anti-mouse β-actin (1:10000, Boster) were added and incubated at 4°C overnight. The membrane was rinsed with PBST for four times, and incubated with HRP labeled secondary antibody for 3h at room temperature. After rinsing with PBST three times, further visualized by exposure to the Image Quant LAS 4000 system (GE Healthcare, Waukesha, USA). β-actin was used as the loading control. The protein bands were quantified densitometrically using Quantity-One protein analysis software (Bio-Rad Laboratories, Hercules, CA, USA).

### Data Collection and Analysis

Data from microarrays GSE53306, which include healthy control and in UC patients, was downloaded from the Gene Expression Omnibus database (GEO database). Differentially expressed Slit2 and Robo1 mRNA levels between UC patients and healthy control groups were identified and analyzed by GEO2R.

### Statistical Analysis

Data are presented as means ± SEM. The data were compared between the two groups by Student *t*-test. P<0.05 was considered statistically significant. Graphs were generated by using GraphPad Prism 8.0 (La Jolla, CA, USA).

## Results

### The expression of Slit2 and Robo1 in colon tissues of patients with UC

**Figure 1:**
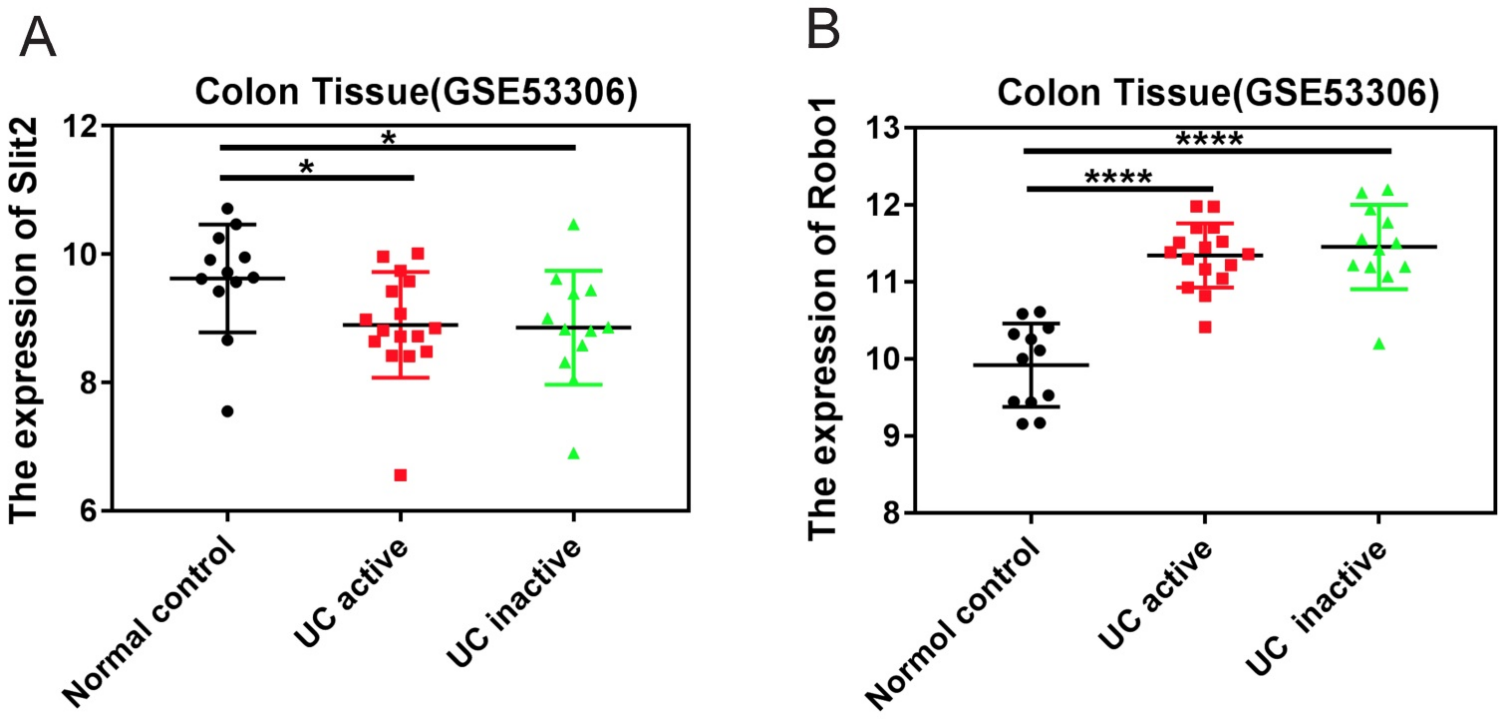
** The expression of Slit2 and Robo1 in patients with UC.** Relative gene expression levels of Slit2 in colon tissue that was sampled during consecutive active and quiescent stages at the same site in individual UC patients. The active and inactive stages were compared with each other as well as with healthy control cases from the GEO database. (A) relative Slit2 expression (n=12) and (B) relative Robo1 expression in the colon tissues of UC patients (n=12) compared with healthy controls (n=24).

The GEO datasets, which is a collection of aggregate array data for deeper analysis of gene expression in disease, was used to analyze the expression of Slit2 and Robo1 in colon tissues of patients with UC. Tissues were sampled during consecutive active and quiescent stages at the same site in individual UC patients. The active and inactive stages were compared with each other as well as to non-inflammatory bowel disease (non-IBD) healthy controls (GSE53306). The expression of Slit2 was decreased in UC patients compared to that of healthy controls (Figure 1A), whereas increased Robo1 levels were detected in UC patients (Figure 1B), suggesting that abnormal Slit2/Robo1 signaling might be involved in the development of pathological processes in UC.

### Slit2/Robo1 signaling is involved in DSS-induced ulcerative colitis

**Figure 2:**
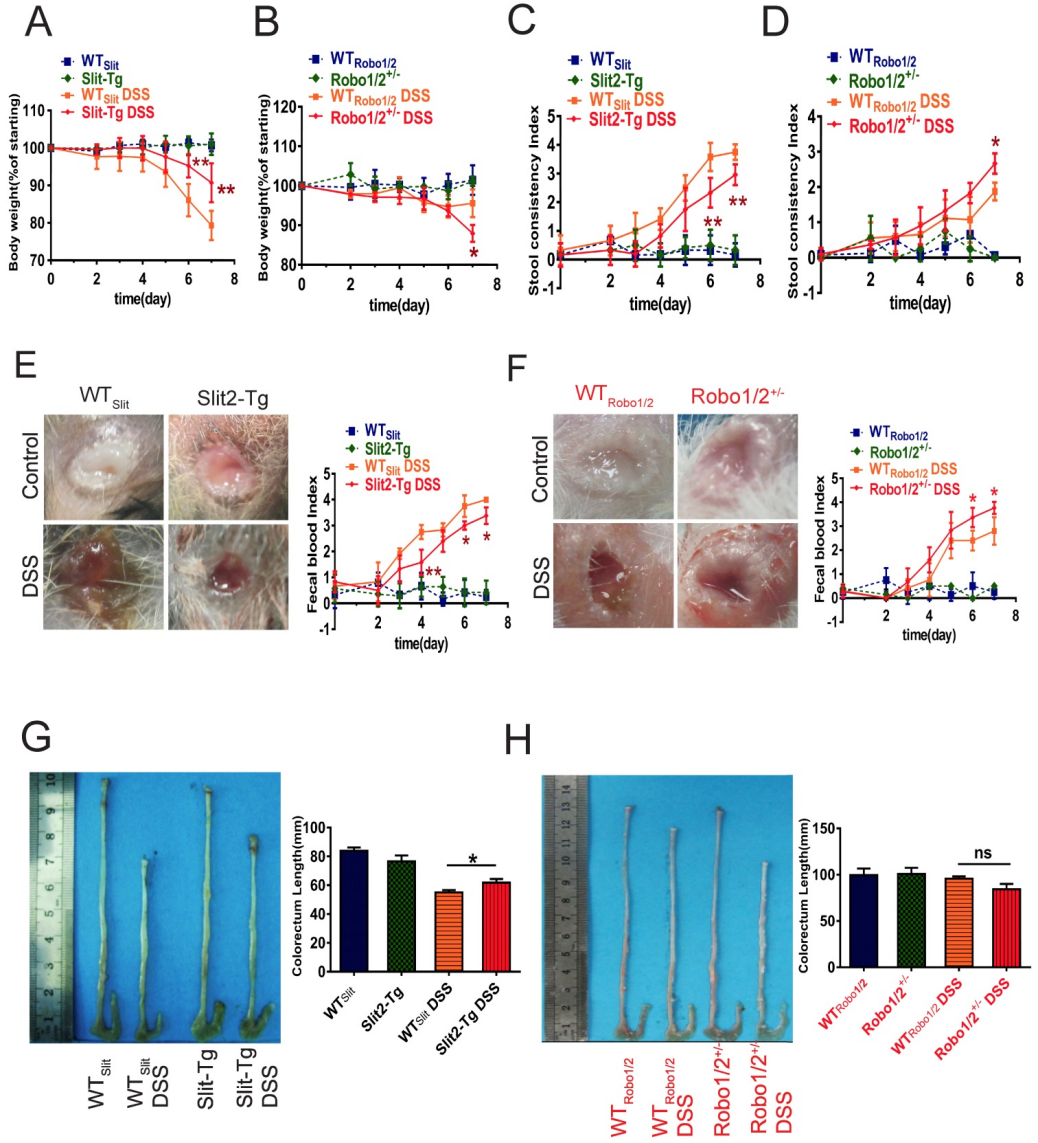
** Physiological changes and intestinal inflammation in mice induced with DSS.** Body weights at different time points in (A) WT_Slit_ and Slit2-Tg mice and (B) WT_Robo1/2_ and Robo1/2^+/-^ mice; Fecal consistency of the mice during the experiment; (C) WT_Slit_ and Slit2-Tg mice and (D) WT _Robo1/2_ and Robo1/2^+/-^ mice; Feces were collected from the colon of individual mice immediately after sacrifice, and blood content was tested in fecal occult blood from (E) WT_Slit_ and Slit2-Tg mice and (F) WT_Robo1/2_ and Robo1/2^+/-^ mice; Representative photographs of the colons at day 7 of DSS-induced and the statistic of colon lengths in (G) WT_Slit_ and Slit2-Tg mice and (H) WT_Robo1/2_ and Robo1/2^+/-^ mice; n=10 in each group. Data are present as means ± SEM.

Based on the GEO analysis, we next investigated whether Slit2/Robo1 signaling affects DSS-induced colitis in Slit2-Tg and Robo1/2 ^+/-^ mice. After the administration of 2% DSS, the body weights of mice in the DSS group at day 7 decreased significantly compared to those of the control group (Supplementary Figure 1). In addition, the degree of fecal occlumency increased while the length of colorectum became shorter. Slit2-Tg mice showed less body weight loss (Figure 2A), lighter stool consistency (Figure 2C), less fecal occult blood (Figure 2E), and less colorectal shortening after DSS administration (Figure 2G) compared to those of WT_Slit_ mice. In contrast, partial knockout of Robo1/2 in mice resulted in more DSS-induced weight loss at day 7 (Figure 2B), increased stool consistency (Figure 2D), increased fecal occult blood (Figure 2F), and slightly colorectal shortening (Figure 2H).

**Figure 3:**
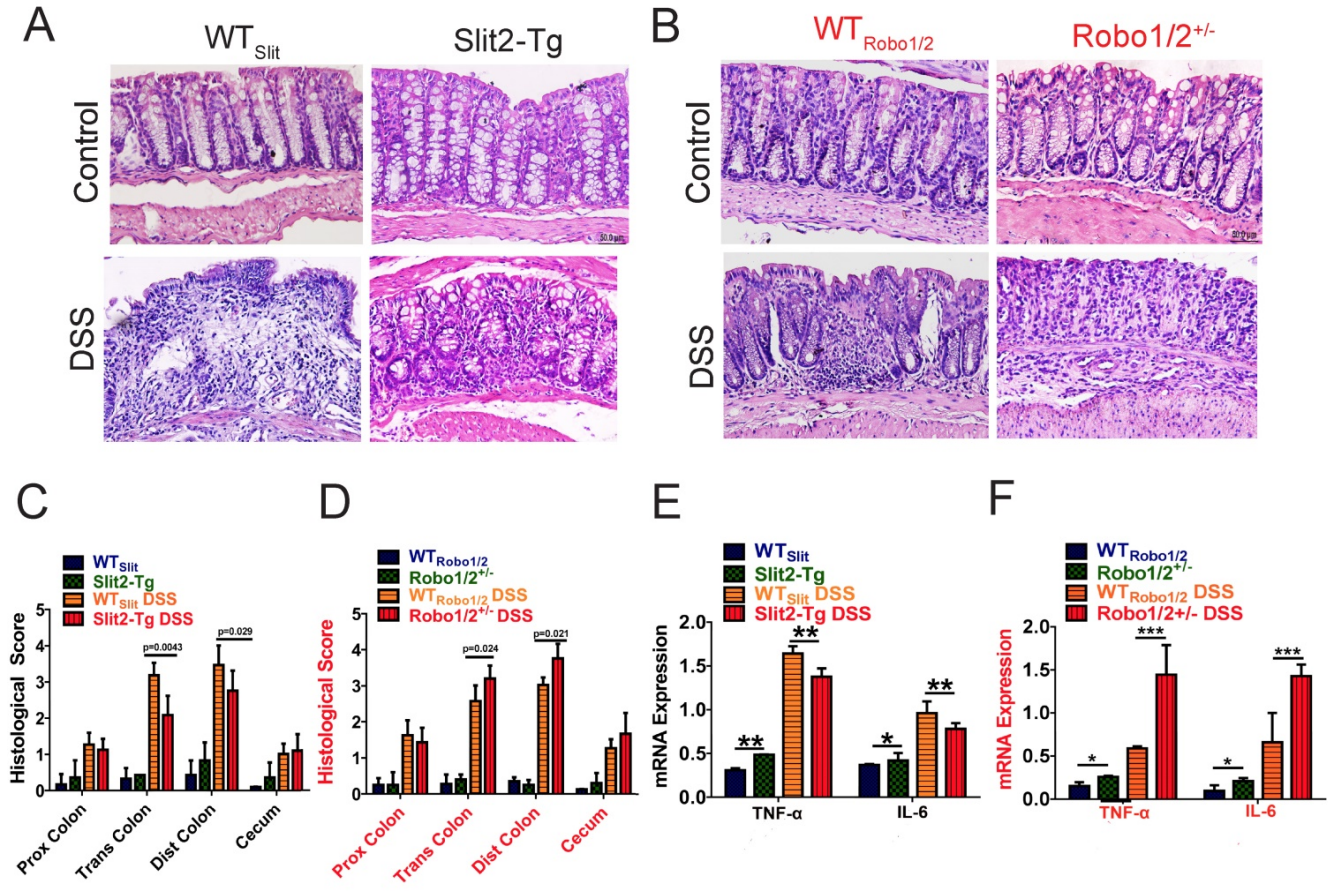
** Histological evaluation of colitis and expression of inflammatory factors in the colon tissue of mice.** Hematoxylin-eosin staining in colon tissue from (A) WT_Slit_ and Slit2-Tg mice and (B) WT_Robo1/2_ and Robo1/2^+/-^ mice; Histological scores of (C) WT_Slit_ and Slit2-Tg mice and (D) WT_Robo1/2_ and Robo1/2^+/-^ mice (n=6-8; scale bar = 50 μm); qRT-PCR measurement of the mRNA expression of IL-6 and TNF-α in the colon of (E) WT_Slit_ and Slit2-Tg mice and (F) WT_Robo1/2_ and Robo1/2^+/-^ mice (n=3). Data are present as means ± SEM. *P<0.05, **P<0.01, ***P<0.001, ****P<0.0001.

Moreover, the DSS group developed apparent inflammation characterized by incompleteness in colonic structure, ulcers, focal necrosis, inflammatory cell infiltration, and partial dilation of gland crypts compared to those of the control group (Figure 3A-B). Sections of colonic tissue from the DSS group stained with H&E presented a significantly higher total histological score than those in the control group (Figure 3C-D). These physiological and pathological features indicate that Slit2 inhibits DSS-induced intestinal ulcerative inflammation, and Robo1/2^+/-^ partial knockout promotes DSS-induced intestinal ulcerative inflammation. Consistently, the mRNA levels of TNF-α and IL-6 in colon tissue of Slit2-Tg DSS mice were lower than those in WT_Slit_ DSS mice (Figure 3E); instead, in Robo1/2^+/-^ mice, the mRNA levels of TNF-α and IL-6 were increased after DSS treatment compared with those of WT_Robo1/2_ mice (Figure 3F).

### Slit2/Robo1 signaling regulated intestinal stem cell proliferation in UC

**Figure 4:**
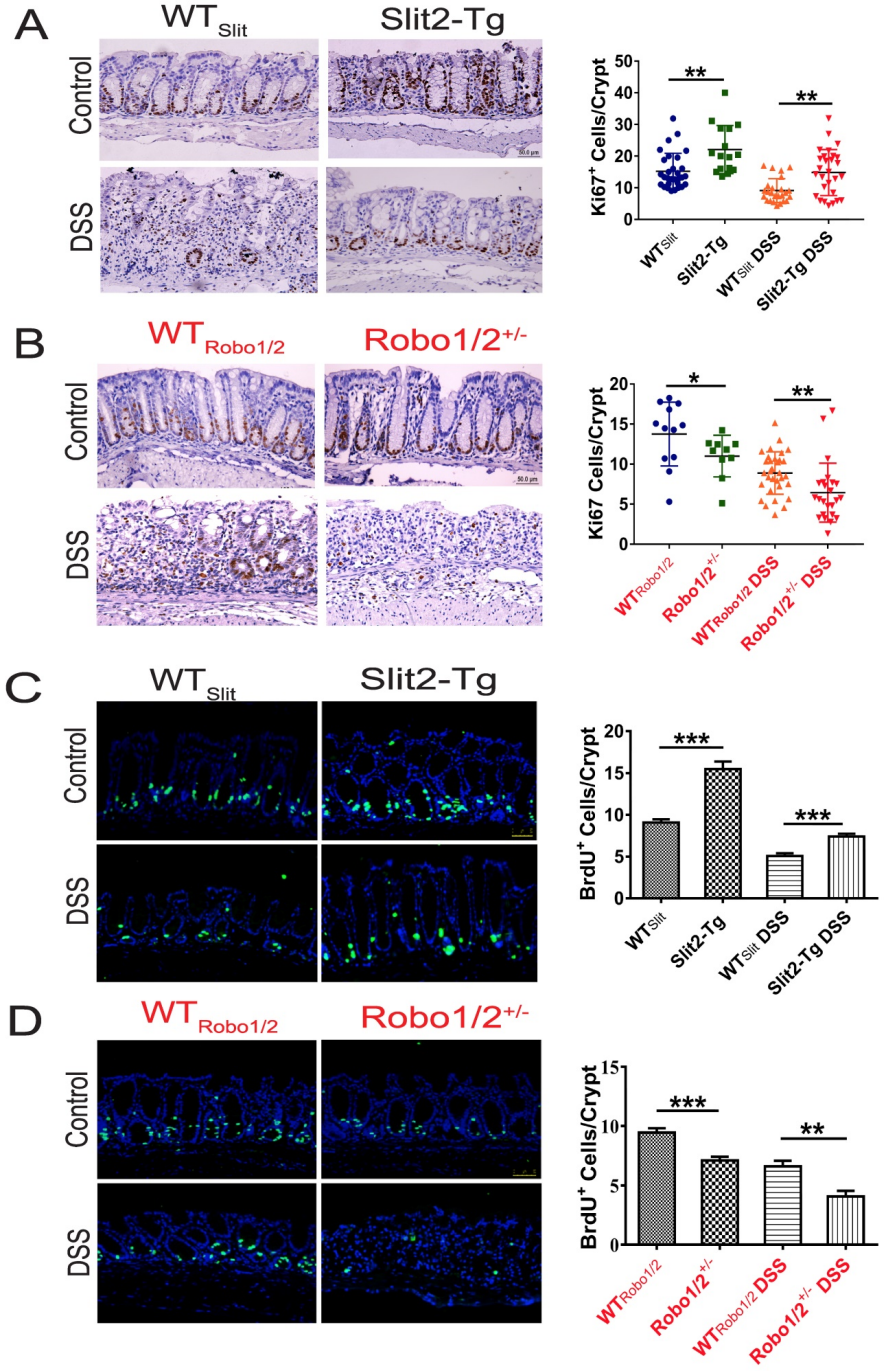
** The proliferation of intestinal stem cell in the colons of DSS-induced mice.** Immunohistochemical staining of Ki67 in colon tissue of (A) WT_Slit_ and Slit2-Tg mice and (B) WT_Robo1/2_ and Robo1/2^+/-^ mice; Immunofluorescence staining of BrdU^+^ in colon tissue of (C) WT_Slit_ and Slit2-Tg mice and (D)WT_Robo1/2_ and Robo1/2^ +/-^ mice; n=6-8 in each group; scale bar = 50 µm. Data are present as means ± SEM. *P<0.05, **P<0.01, ***P<0.001.

**Figure 5:**
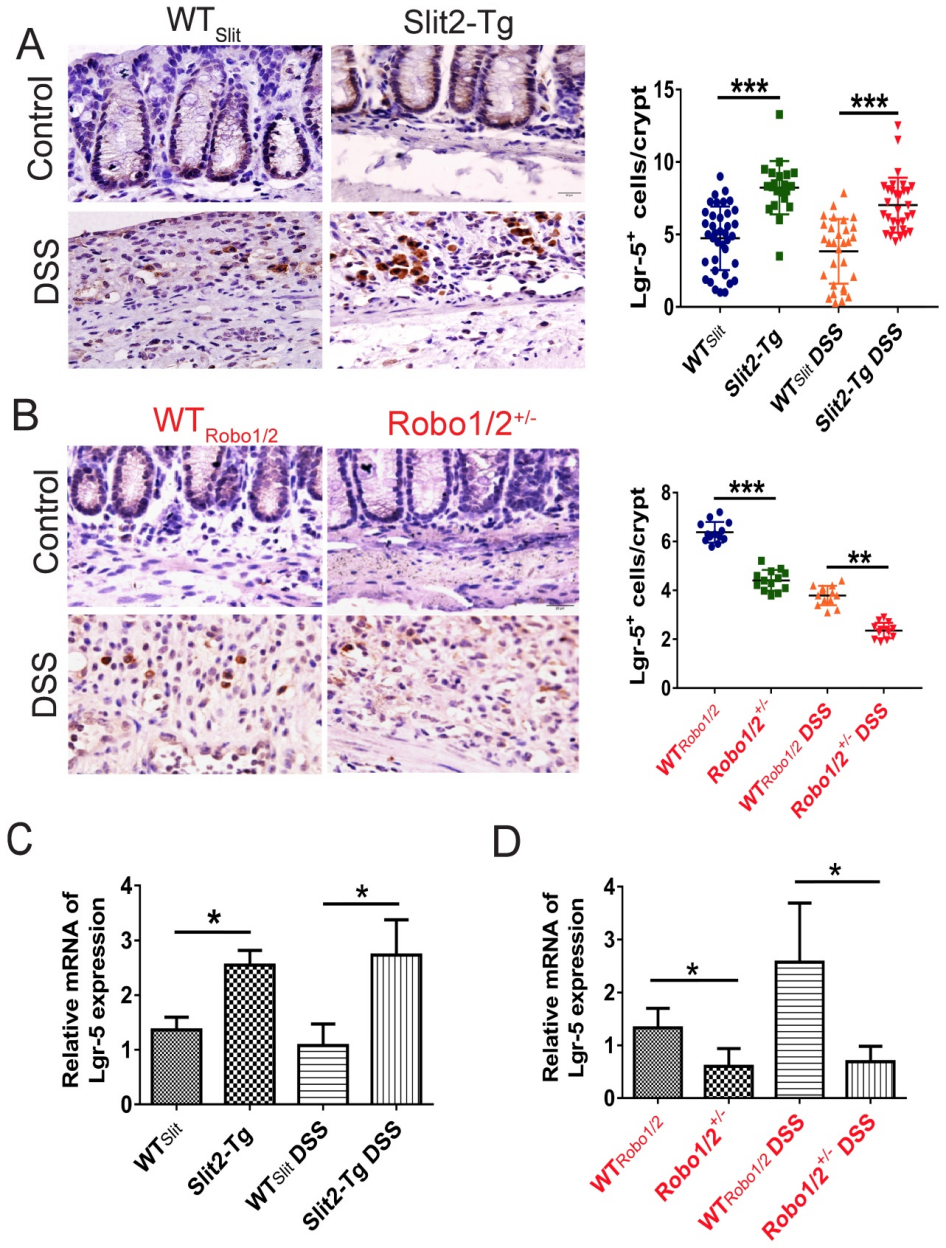
** The expression of Lgr5 in the colon tissue of DSS-induced mice.** Immunohistochemical staining of Lgr5^+^ in colon tissue of (A) WT_Slit_ and Slit2-Tg mice and (B) WT_Robo1/2_ and Robo1/2^+/-^ mice (n=6 in each group, scale bar = 20 μm); The mRNA expression of Lgr5 as assessed by RT-qPCR in (C) WT_Slit_ and Slit2-Tg mice and (D) WT_Robo1/2_ and Robo1/2^+/-^ mice (n=3 in each group); data are present as means ± SEM. *P<0.05, **P<0.01, ***P<0.001.

Intestinal stem cells (ISCs) 27 expressed Slit2 and its single-span transmembrane cell-surface receptor Robo1 28. A recent study showed that a Slit2 transgene increased ISC numbers where partial genetic deletion of Robo1 decreased ISC numbers 26. Furthermore, Lgr5^+^ cells colocalized with Slit2 and Robo1 in the colon crypts of Slit2-Tg and Robo1/2^+/-^ mice 26. Considering Slit2 transgene mice mitigated inflammation in colon tissue induced by DSS, we further examined the effect of Slit2/Robo1 on the ISCs proliferation after DSS treatment. In the intestinal structure, stem cells are distributed at the bottom of the crypt, and transient amplifying (TA) is distributed in the middle of the crypt. In our immunohistochemistry results, the number of Ki67 and BrdU^+^ cells in the Slit2-Tg DSS group was higher in the colon crypt than that in the WT_Slit_ DSS group (Figure 4A, C), whereas, in Robo1/2^+/-^ DSS mice, Ki67 and BrdU^+^ cell numbers decreased compared with that of WT_Robo1/2_ mice (Figure 4B, D). Besides, immunohistochemistry analysis showed a greater number of Lgr5^+^ stem cells in the Slit2-Tg DSS mice than in WT_Slit_ DSS mice (Figure 5A), whereas Robo1/2 partial knockout decreased the number of Lgr5^+^ stem cells (Figure 5B). Moreover, qPCR assays showed that the expression of Lgr5 was higher in Slit2-Tg DSS mice than in WT_Slit_ DSS mice (Figure 5C). Instead, the level of Lgr5 in the intestinal tissue of Robo1/2^+/-^ DSS mice was lower than that in WT_Robo1/2_ mice (Figure 5D), indicating that Slit2 overexpression maintained ISCs self-renewal function after DSS treatment and therefore reduced colon epithelium injured compared with WT_slit_ mice whereas partial Robo1/2 knockout promoted ISCs reduction induced by DSS compared with WT_Robo1/2_ mice.

### The Slit2/Robo signaling modulated the expression of autophagy proteins in the crypt

Autophagy has been suggested to regulate the maintenance, expansion, and differentiation of stem cells 21. Given the unique capacity of ISCs of Slit2-Tg mice in remaining themselves proliferation in the UC process, it is predicted that autophagy may function to balance the self-renewal and the quiescence. Therefore, we detected whether Slit2/Robo1 impacted the autophagy in ISCs.

**Figure 6:**
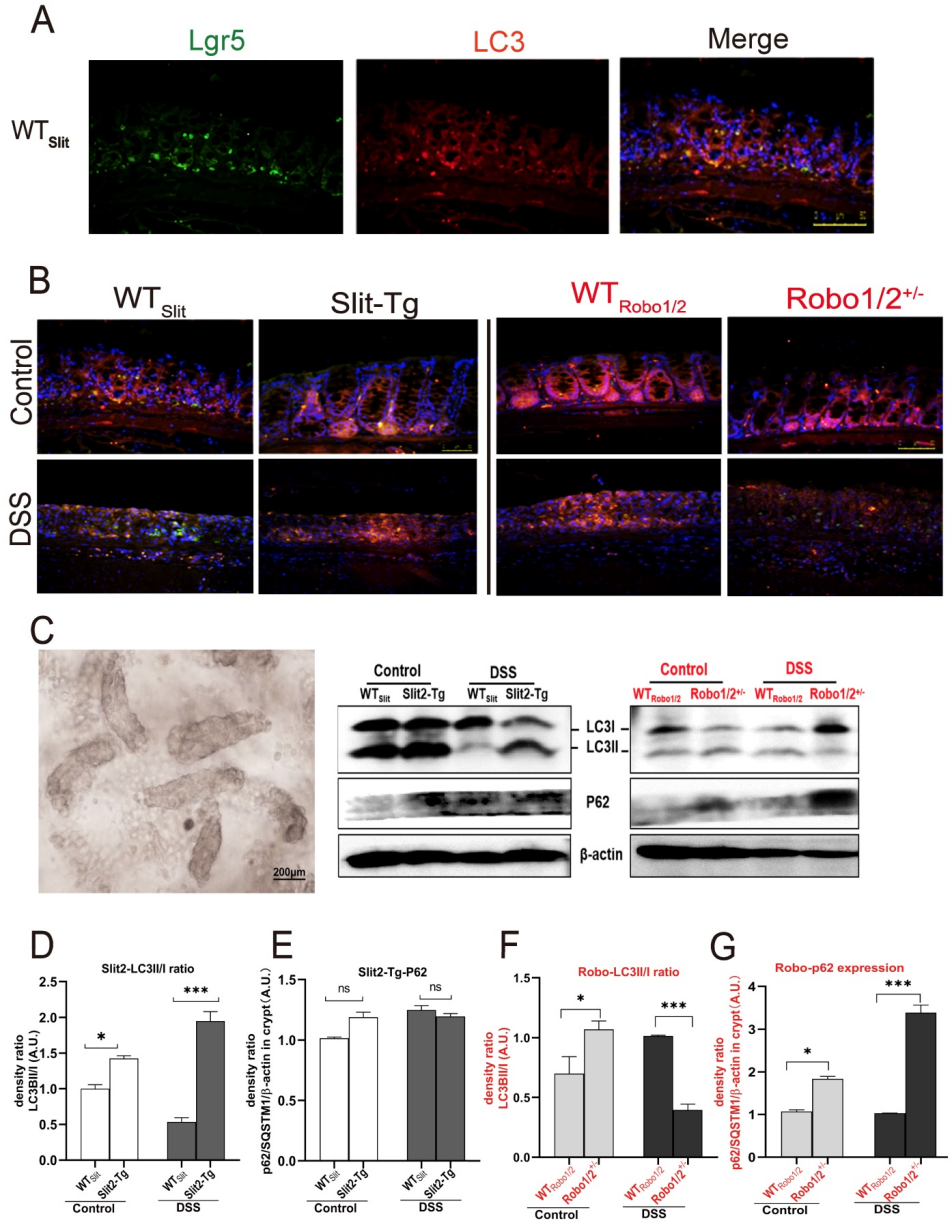
** Expression of the autophagy proteins in DSS-induced mice.** The coexpression of Lgr5^+^ colonic stem cells and the autophagy protein LC3 in (A) WT_Slit_ and Slit2-Tg mice and (B) WT_Robo1/2_ and Robo1/2^+/-^ mice. Lgr5 (green) and LC3 (red) expression in the colonic epithelium. Sections were counterstained with DAPI (blue). (n=5 in each group; scale bar = 50 μm); (C) the photograph of the crypt isolated from colon tissue (scale bar= 200μm); (D) protein expression of LC3II/I in (D) WT_Slit_ and Slit2-Tg mice and (F) WT_Robo1/2_ and Robo1/2 ^+/-^ mice (n=4); The expression of P62 in (E) WT_Slit_ and Slit2-Tg mice and (G) WT_Robo1/2_ and Robo1/2^+/-^ mice (n=3-4 in each group). Detection of β-actin served as a loading control. Quantification of bands is expressed as density ratio of indicated protein/β-actin (A.U.); data are present as means ± SEM. *P<0.05, **P<0.01, ***P<0.001.

In higher eukaryotes, autophagy initiation, cargo recognition, cargo engulfment, and vesicle closure are LC3/GABARAP protein family protein dependent. LC3 is associated with autophagosome development and maturation and is used to monitor autophagic activity 29. We observed that LC3 mainly colocalized with Lgr5^+^ stem cell in the base of colonic crypts of mice with different genotypes (Figure 6A-B), suggesting that Slit2/Robo1 probably modulate autophagy in Lgr5^+^ intestinal stem cells. Furthermore, we isolated the crypts (Figure 6C) from the colon to enrich Lgr5^+^ stem cells and quantified the expression of the autophagy proteins. LC3I is present in cytoplasmic, and the formation of membrane-bound isoforms LC3II complexes is a feature of functional autophagosomes. The LC3II/LC3I ratio (LC3II/I ratio), used as an indicator of autophagic activation. p62/SQSTM1 is a multifunctional protein and a marker of the autophagic degradation process. LC3II protein and the ratio of LC3II/I increased in Slit2-Tg mice compared with WT_slit_ mice (Figure 6D). Although the LC3II expression decreased after DSS treatment in WT_slit_ and Slit2-Tg mice, the ratio of LC3II/I markedly elevated in Slit2-Tg mice (Figure 6D), and p62 protein only slightly increased in DSS-treated mice (Figure 6E), suggesting enhancement of autophagy in the Slit2-Tg crypts. In contrast, LC3II protein and the ratio of LC3II/I remarkably decreased (Figure 6F), while p62 protein significantly increased in Robo1/2^+/-^ DSS mice compared with WT_Robo1/2_ DSS mice (Figure 6G), indicating functional deletion of autophagy in Robo1/2^+/-^ crypts.

## Discussion

In the present study, we first reported that Slit2 overexpression alleviated inflammatory injury and the expression of proinflammatory cytokines in the colon tissue induced by DSS. Meanwhile, Robo1/2 partial knockout exacerbated pathological changes. Furthermore, Slit2 overexpression maintained the proliferation and normal autophagy flux in Lgr5^+^ stem cell after DSS treatment while Robo1/2 partial knockout reduced the number of Lgr5^+^ stem cell and retarded the autophagy flux in Lgr5^+^ stem cell, suggesting Slit2/Robo1 mitigated DSS-induced UC probably via regulation of autophagy in Lgr5^+^ ISCs. Besides, the GEO database 30 analysis showed that abnormal expression of Slit2/Robo1 in the colon tissue of UC patients compared with that of healthy people.

UC is a chronic inflammatory disorder of the gastrointestinal tract 31. The abnormal immune response or destruction of normal immune regulation is an important part of the pathogenesis of UC 32, 33. Overproduction of proinflammatory cytokines can produce chemotactic effects on inflammatory cells, including neutrophils, to attract them into intestinal lesions, resulting in intestinal mucosal edema, increased permeability of the intestinal mucosa and an exacerbated intestinal reaction, eventually leading to the occurrence of UC 7, 34. Histopathological examination of colon sections showed more severe epithelial erosion, areas of mucosal ulceration, and increased numbers of infiltrating mucosal and submucosal leukocytes in Robo1/2^+/-^DSS mice compared with those of their control littermates, resulting in higher histological scores for both tissue damage and inflammation. However, the results in Slit2-Tg DSS mice were the opposite. Besides, Slit2 overexpression decreased the mRNA level of TNF-α and IL-6 in the colon tissue induced by DSS compared with that of WT_Slit_ mice. In contrast, Robo1/2 partial knockout stimulated the mRNA expression of TNF-α and IL-6, suggesting Slit2/Robo1 signals involved in the development of UC.

Of note, our findings showed that Slit2 overexpression maintained the integrity of colon epithelium after DSS treatment, which is related to the activity of Lgr5^+^ stem cell that possesses a remarkable self-renewal ability. The crypt base columnar cells marked by Lgr5 mediated the daily renewal of intestinal epithelium 35, 36. Additionally, endogenous Slit2 and Robo1 only expressed in Lgr5^+^ stem cells in the intestinal crypt and promoted Lgr5^+^ stem cells differentiated into epithelial cells.

We observed that with DSS administration, the number of crypt cells in colon tissue stained with proliferating marker Ki67^+^ and BrdU in Slit2-Tg mice is much higher than that in WT_slit_ mice. In contrast, partial Robo1/2 knockout significantly reduced the number of proliferating cells in the crypt compared with WT_Robo1/2_ mice, suggesting that activation of Slit2/Robo1 probably maintained the function of Lgr5^+^ stem cells after DSS treatment.

Autophagy is an essential mechanism of stem cell maintenance and self-renewal and participates in various stem cell differentiation processes. Besides, defective autophagy has been strongly linked to IBD pathogenesis 37. Macroautophagy (often referred to as autophagy) is a highly conserved bulk protein degradation pathway in eukaryotes. During this process, the membranous cellular structures respond to stimuli by generating a double-membrane structure called the autophagosome, which engulfed the cytoplasmic portions and organelles. Then the autophagosome fuses with the lysosomes to degrade the sequestered materials by various lysosomal hydrolytic enzymes. Activation and successful processing of the autophagic pathway increases LC3-II complexes, indicating the formation of functional autophagosomes 38. However, loss of autophagy causes cytoplasmic accumulation of ubiquitin-positive proteinaceous inclusions. The ubiquitin- and LC3-binding protein p62 regulates the formation of protein aggregates and is removed by autophagy, which plays a vital role in inclusion body formation associated with autophagic deficiency 39. Activation of autophagy reduces the protein expression of p62/SQSTM1 typically, while pharmacological or genetic inhibition of autophagy increases intracellular p62/SQSTM1 levels 40. Here, immunofluorescent assays confirmed the colocalization of LC3 and Lgr5^+^ stem cells in the colon crypt.

Interestingly, after collection of the crypt from colon tissues, Western blotting assay first showed that the ratio LC3II/I increased in Slit2-Tg DSS mice and decreased in Robo1/2^+/-^ DSS mice compared with those in their respective WT littermates. Meanwhile, p62, the cargo receptor, markedly increased in Robo1/2^+/-^ DSS mice and slightly increased in Slit2-Tg DSS mice compared to their respective WT littermates. Slit2 overexpression might promote normal autophagy process with little p62 protein deposition, while partial Robo1/2 knockout probably stops the autophagy and accumulate a high level of p62 protein in crypt cells. These findings indicated the crucial role of Slit2/Robo1 signals in maintaining Lgr5^+^ stem cell proliferation via regulating autophagy in ISCs.

However, there is still a limitation to this study. It is unknown how Slit2/Robo1 signaling mediates the autophagy process. If possible, Lgr5^+^ stem cells must be isolated from both types of mice and treated with autophagy inhibitors such as rapamycin or 3-MA to confirm and investigate the mechanism by which Slit2/Robo1 modulated the process of autophagy in the intestinal stem cells in the future.

## Conclusions

This study first observed that Slit2/Robo1 signaling plays a vital role in the occurrence and development of UC and probably involved in modulating the autophagy process in Lgr5^+^ stem cells, which may be a new target for the development of drugs to treat inflammatory intestinal diseases.

## Supplementary Material

Supplementary figure.
