# Supplementary material for: Slit2/Robo1 Mitigates DSS-induced Ulcerative Colitis by Activating Autophagy in Intestinal Stem Cell
Source: Int J Biol Sci. 2020 Apr 6;16(11):1876–87. doi: 10.7150/ijbs.42331 (PMC7211176; doi:10.7150/ijbs.42331)
Supplement: Supplementary file 1 — Supplementary figure. [file ijbsv16p1876s1.pdf]

Supplemental figure 1. The body weight of $WT_{Robo1/2}$ and $Robo1/2^{+/-}$ mice at Day 7 after DSS treatment. n=10 in each group; data are present as means ± SEM. *P<0.05, **P<0.01.

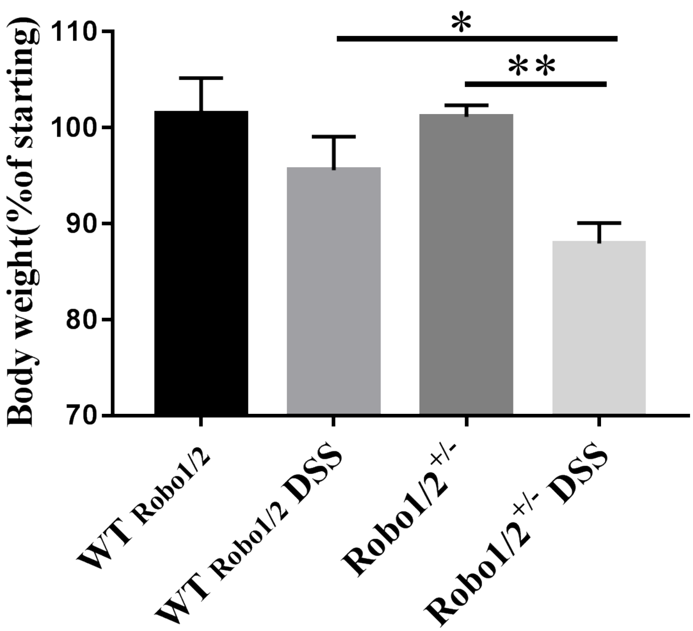

Body weight(%of starting)
110
100
90
80
70
*
**
WT Robo1/2
WT Robo1/2 DSS
Robo1/2+/-
Robo1/2+/- DSS
